# Telemedical management in patients waiting for transcatheter aortic valve implantation: the ResKriVer-TAVI study design

**DOI:** 10.3389/fcvm.2023.1352592

**Published:** 2024-01-22

**Authors:** Isabel Mattig, Kerstin Koehler, Gina Barzen, Meike Hiddemann, Elias Kugel, Constantin Roemmelt, Verena Mauckisch, Clarissa Vockeroth, Karl Stangl, Thomas Hoppe, Friedrich Koehler, Henryk Dreger

**Affiliations:** ^1^ Deutsches Herzzentrum der Charité, Department of Cardiology, Angiology and Intensive Care Medicine, Campus Charité Mitte, Berlin, Germany; ^2^ Charité - Universitätsmedizin Berlin, Corporate Member of Freie Universität Berlin and Humboldt-Universität zu Berlin, Berlin, Germany; ^3^ DZHK (German Centre for Cardiovascular Research), Partner Site Berlin, Berlin, Germany; ^4^ Berlin Institute of Health at Charité - Universitätsmedizin Berlin, BIH Biomedical Innovation Academy, Berlin, Germany; ^5^ Charité - Universitätsmedizin Berlin, Corporate Member of Freie Universität Berlin, Humboldt-Universität zu Berlin, and Berlin Institute of Health, Medical Department, Division of Cardiology and Angiology, Centre for Cardiovascular Telemedicine, Berlin, Germany; ^6^ Fraunhofer FOKUS, Berlin, Germany; ^7^ Deutsches Herzzentrum der Charité, Department of Cardiology, Angiology and Intensive Care Medicine, Campus Virchow Klinikum, Berlin, Germany

**Keywords:** aortic stenosis, transcatheter aortic valve implantation, TAVI, COVID-19 pandemic, telemedical interventional management

## Abstract

**Aims:**

The majority of patients with severe aortic stenosis (AS) planned for transcatheter aortic valve implantation (TAVI) are elective outpatients. During the COVID-19 pandemic, the time between the heart team’s decision and TAVI increased due to limited healthcare resources. We therefore implemented telemedical approaches to identify AS patients at risk for clinical deterioration during the waiting time. The purpose of the prospective, randomized, controlled ResKriVer-TAVI study (DRKS00027842) is to investigate whether a digital concept of telemedical interventional management (TIM) in AS patients waiting for TAVI improves the clinical outcomes. In the present article, we report the study protocol of the ResKriVer-TAVI trial.

**Methods:**

ResKriVer-TAVI will enroll AS patients planned for elective TAVI. Randomization to the TIM group or standard care will be made on the day of the heart team’s decision. TIM will include a daily assessment of weight, blood pressure, a 2-channel electrocardiogram, peripheral capillary oxygen saturation, and a self-rated health status until admission for TAVI. TIM will allow optimization of medical therapy or an earlier admission for TAVI if needed. Standard care will not include any additional support for patients with AS. All patients of the TIM group will receive a rule-based TIM including standard operating procedures when a patient is crossing prespecified values of a vital sign.

**Results:**

The primary endpoint consists of days lost due to cardiovascular hospitalization and death of any cause within 180 days after the heart team’s decision. Major secondary endpoints include all-cause mortality within 365 days, the number of telemedical interventions, and adherence to TIM. Follow-up visits will be conducted at admission for TAVI as well as 6 and 12 months after the heart team’s decision.

**Conclusions:**

ResKriVer-TAVI will be the first randomized, controlled trial investigating a telemedical approach before TAVI in patients with AS. We hypothesize that primary and secondary endpoints of AS patients with TIM will be superior to standard care. The study will serve to establish TIM in the clinical routine and to increase the resilience of TAVI centers in situations with limited healthcare resources.

## Introduction

Moderate to severe aortic stenosis (AS) is one of the most prevalent valvular heart diseases in the elderly, reaching a prevalence of 2.8% in patients aged ≥75 years (1). AS is associated with high morbidity and mortality; the mean survival of patients with untreated severe AS is 2 years (2). According to current guidelines, therapy of AS comprises surgical or transcatheter aortic valve implantation (TAVI) (3). In recent years, a growing number of TAVI procedures have been performed globally and, according to current recommendations, the indication for TAVI has been extended to younger and low-risk patients (3, 4). This was associated with an increase in waiting times for elective AS outpatients (5). In many centers, the waiting time between the heart team's decision and intervention exceeds 1 month. In addition, the COVID-19 pandemic resulted in limited resources also affecting TAVI procedures (6). During this period, many patients with AS stayed untreated at home and were kept on a waiting list. TAVIs were performed according to symptoms and severity of AS versus available hospital resources (7). Albassam et al. observed a mortality of approximately 5% during a median waiting period of 84 days (8). In addition to reported mortality, untreated severe AS may exacerbate and lead to decompensated heart failure (9).

Telemedical interventional management (TIM) may reduce morbidity and mortality among patients with AS. TIM, including a wide range of devices, is well studied as an outpatient management of patients with heart failure (10–14). In the TIM-HF2 trial, the remote patient management (RPM) comprised non-invasive TIM, treatment of heart failure and comorbidities, close cooperation with primary care physicians, and patient education, and was used for a maximum follow-up period of 393 days (15). In this specific patient cohort, the RPM reduced days lost to unplanned cardiovascular hospitalizations and all-cause deaths compared to standard care (15).

Despite a lack of evidence, national cardiac societies recommended the establishment of TIM in valvular heart disease during the COVID-19 pandemic (7, 16). Comprehensive TIM may address the healthcare gap during the waiting period between the day of the heart team's decision to inpatient admission for TAVI in patients with AS in times of limited healthcare resources but also under normal conditions when waiting times are long due to increased demand. Therefore, we implemented telemedical approaches to identify AS patients at risk for clinical deterioration during the waiting time. The purpose of the ResKriVer-TAVI study is to investigate whether a digital concept of telemedical interventional management (TIM) in AS patients waiting for a TAVI improves the clinical outcome of these patients. Here, we report the study protocol of the ResKriVer-TAVI trial.

## Methods

The ResKriVer-TAVI study (DRKS00027842) is a single-center, prospective, parallel-group randomized, controlled, superior, open-label trial started in 2022 at the Charité—Universitätsmedizin Berlin, Germany, and complies with the Declaration of Helsinki. The study is approved by the institutional ethics committee (EA1/017/22) and financially supported by the German Federal Ministry for Economic Affairs and Climate Action, Berlin, Germany.

We will enroll patients with AS recommended to undergo TAVI by our local heart team. AS will be defined and assessed according to the guidelines of the European Society of Cardiology (ESC) and the European Association for Cardio-Thoracic Surgery (EACTS) (3). Patients with an inability or unwillingness to measure daily vital signs (e.g., dementia, blindness, deafness, or wheelchair use) and patients enrolled in other interventional trials will be excluded from the study. Patients with AS will be screened for eligibility during their outpatient visit or hospital stay for AS assessment followed by a baseline visit after the patients have provided signed informed consent. Diagnostic work-up to grade AS and evaluate patients for TAVI or surgical approach will include medical history, laboratory measurements, echocardiography, electrocardiogram, coronary angiography, and computed tomography as a part of clinical routine. On the day of the heart team’s decision, patients will be randomized to TIM and standard care or standard care alone. Standard care will not include any additional support for patients with AS who are on the waiting list. Stratified randomization will be performed using an automatic randomization tool considering three covariates: male vs. female, age ≤80 years vs. age >80 years, and NYHA class ≤ II vs. NYHA class ≥ III. Data management will be completed in electronic case report forms.

### Study objective

**Figure 1:**
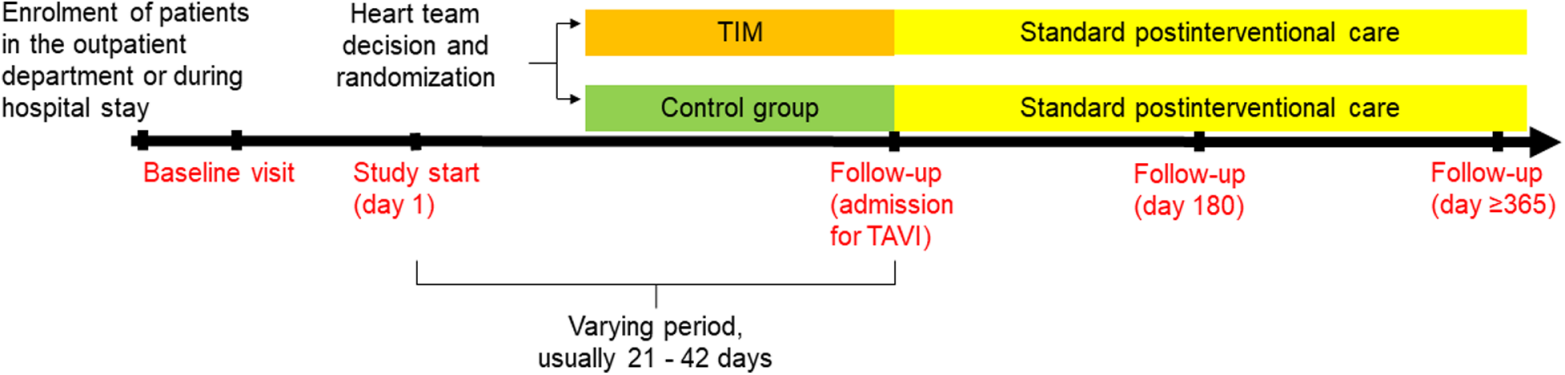
Flow chart of the ResKriVer-TAVI study. TAVI, transcatheter aortic valve implantation; TIM, telemedical interventional management.

We hypothesize that patients with AS who are monitored by TIM during the waiting period will demonstrate significantly fewer days lost to cardiovascular hospitalization and all-cause mortality compared to standard care during the 180 days after the heart team's decision (primary endpoint). Secondary endpoints are listed in Table 1. Follow-up visits are planned at admission for TAVI, 180 days, and 365 days after the heart team's decision (Figure 1). The study duration for each patient will be 365 days after the heart team's decision (day 1-day 180: assessment of primary and secondary endpoints, day 181-day 365 evaluation of survival status).

**Table 1:** Primary and secondary endpoints of the ResKriVer-TAVI study.

Primary endpoint
• Days lost to cardiovascular hospitalization and all-cause mortality 180 days after the heart team's decision
Secondary endpoints
• Days lost to cardiovascular hospitalization 180 days after the heart team's decision • Days lost to all-cause mortality 180 days after the heart team's decision • All-cause mortality 180 days after the heart team's decision • All-cause mortality 365 days after the heart team's decision • Number of unplanned cardiovascular hospitalizations 180 days after the heart team's decision • Number of hospitalizations due to heart failure decompensation 180 days after the heart team's decision • Number of interventions by TIM • Number of hospital admissions as recommended by telemedical physicians and nurses • Patients’ adherence to TIM • Postponement of TAVI due to medical reasons (reported in days) • Quality of life assessed by PROMIS-29 + 2 questionnaire (at baseline, admission for TAVI, 180 days after heart team decision) • NYHA class (at baseline, admission for TAVI, 180 days after heart team decision) • Laboratory measurements including GFR (at baseline, admission for TAVI, 180 days after heart team decision)

TIM, telemedical interventional management; TAVI, transcatheter aortic valve implantation; PROMIS, patient-reported outcomes measurement information system (17, 18); NYHA class, New York Heart Association class; GFR, glomerular filtration rate.

### Intervention

**Figure 2:**
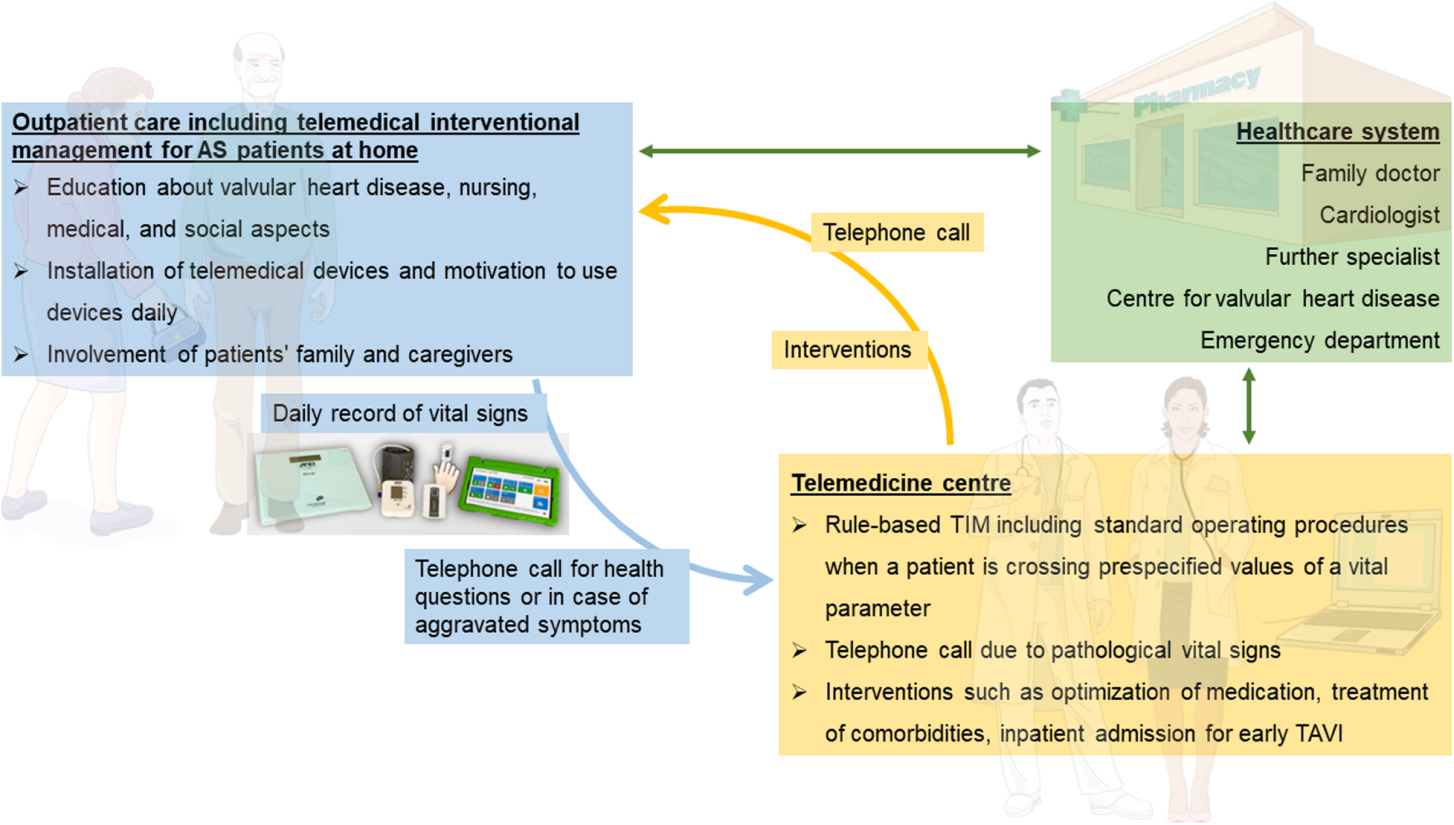
Overview of telemedical interventional management at patients’ homes (purple box), telemedicine center (orange box), and their interaction with the healthcare system (blue box). AS, aortic stenosis; TAVI, transcatheter aortic valve implantation. Parts of the figure were drawn by using pictures from Servier Medical Art. Servier Medical Art by Servier is licensed under a Creative Commons Attribution 3.0 Unported License (https://creativecommons.org/licenses/by/3.0/).

After randomization to the TIM group, telemedical devices will be installed by specialized nurses in patients’ homes, and measurements will be performed daily until hospital admission for TAVI. Therefore, the duration of TIM will be a varying period of approximately 21–42 days. TIM includes three sections: patient education, telemonitoring of vital signs, and telemedical interventions (Figure 2).

Patient education, performed by specialized nurses, has the aim to impart knowledge in the use of telemedical devices and the patient's disease. Patients receive the TIM devices at the beginning of monitoring and are trained regarding their use and benefits. Patients will be motivated to record vital signs every day, which is supported by a manual on how to perform measurements. Moreover, information on valvular heart disease, including possible symptoms, medical consultation, and behavior in an emergency, but also on nursing, medical, and social aspects will be provided. Examples of training include instruction on devices, guidance on medication and fluid intake, as well as lifestyle modifications tailored to specific questions and needs of the patient, and will be performed using a structured script. Additionally, a nursing assessment form will be utilized to determine the necessity for assistance and the potential provision of aids such as a wheelchair. This will be followed by advice on preventative programs and homecare; if necessary, required information on further care will be provided and external support services will be arranged. The education will involve the patient's family and caregivers.

All patients of the TIM group will receive a rule-based TIM including standard operating procedures when a patient is crossing prespecified values of vital signs (Table 2). Telemonitoring of vital signs will comprise a daily assessment of weight (A&D Precision Health Scale Model UC-352BLE, Oxfordshire, OX14 1DY, United Kingdom), blood pressure (A&D UA-651BLE Medical blood pressure monitor, Oxfordshire, OX14 1DY, United Kingdom), a 2-channel electrocardiogram (PhysioMem® PM 100, Gleisdorf, Austria), peripheral capillary oxygen saturation (Beurer PO 60 Bluetooth, Fürth, Germany), and a self-rated health status using a tablet. The self-rated health status includes a standardized scale ranging from “much worse” to “much better” (six grades), providing patients with a simple and easily understandable categorization. The medical devices are provided by GETEMED (Teltow, Germany), validated for telemedical use, and CE-certified. Data will be transmitted securely every day via mobile network, transferred to electronic patient files of the telemedicine center, and evaluated by experienced physicians and nurses. Patients with vital signs out of range will be assessed first and pathological parameters will be validated by a telephone call.

**Table 2:** Prespecified values of vital signs used for TIM.

Heart rate	<50 bpm or >100 bpm
Systolic blood pressure	<90 mmHg or >140 mmHg
Diastolic blood pressure	<40 mmHg or >90 mmHg
Weight gain	>1 kg within one day
	>2 kg within three days
	>2.5 kg within eight days
Oxygen saturation	<94%
Health status	Statement of the patient that his/her condition is “worse” or “much worse” compared to the previous day

Values can be adjusted individually for each patient. TIM, telemedical interventional management.

Analysis of daily vital signs forms the basis for telemedical interventions. In the case of pathological values, recommendations and interventions will be executed depending on the severity of symptoms. Interventions may involve adjusting medication and optimizing therapy for comorbidities in accordance with current guidelines. Moreover, physicians can refer patients to a specialist at an outpatient or emergency department or admit patients earlier for TAVI due to symptom progression. Examples of medical adjustments include changes in antihypertensive or diuretic treatment, often in collaboration with the patient's primary care physician. Additionally, patients will be able to contact the telemedicine center by phone in case of emergencies or medical issues on a daily basis (from 8:00 a.m. to 4:00 p.m.). Subsequently, expert medical advice will be provided, and if necessary, appropriate measures will be initiated. Vital data will be regularly assessed to determine whether TAVI treatment can be individually delayed for each patient or if there is a need for it to be expedited.

### Statistical analysis

Overall, we plan to enroll 200 patients with AS according to the number of TAVI procedures performed at the Deutsches Herzzentrum der Charité (DHZC), Berlin, and the calculated statistical analysis throughout a study period of 2 years. In detail, 1,250 TAVIs are performed at DHZC annually, 370 of these are treated by the team of the Department of Cardiology, Angiology, and Intensive Care Medicine (the primary enrolling center)—including urgent (∼30%) and elective cases (∼70%). Only elective patients will be eligible for the study. Additionally, we are approximating an enrolment rate of 50%–75%. A difference of 6.7 days in lost days between the intervention group and the standard care group is estimated to be clinically significant and can be reached for the primary endpoint with a two-sided *t*-test, a standard deviation of 16.9, an alpha of 0.05, and a power of 0.8 in a study cohort of 200 patients. This is a sufficiently large and realistic effect.

The primary endpoint will be adjusted for stratification variables of randomization (sex, age, and NYHA class) using ANCOVA with lost days as a dependent variable and intervention group as the main effect. Adjusted means with 95% confidence intervals will be reported. This analysis will have an increased power compared to the *t* test. The primary analysis will be conducted in the full analysis set according to the intention-to-treat principle. If the requirements for parametric analysis are not met, the primary endpoint will be evaluated non-parametrically (Friedman test, rank-based ANOVA). All other endpoints will be analyzed according to their distribution with adequate tests, and estimators will be calculated with a 95% confidence interval. An exploratory analysis will be conducted.

## Discussion

The ResKriVer-TAVI study will be the first prospective, randomized, controlled trial to evaluate the impact of TIM on the outcome of AS patients undergoing TAVI in comparison to standard care. The main objective is to shorten the length of hospital stay to perform TAVI and to avoid hospitalization as well as mortality during the waiting period and after TAVI. A comprehensive TIM, including patient education, daily assessment of vital signs, and telemedical interventions, e.g., adjustment of medication or admission for early TAVI, will enable intensive outpatient care from the day of the heart team's decision until admission for TAVI. Patient education with a focus on AS and the involvement of family and caregivers allows self-reliant management. A strict modification of drug therapy is possible due to daily measurement of vital signs and, if necessary, telephone calls. The telehealth approach may reduce morbidity and mortality in case of limited healthcare resources but may also create optimal patient conditions prior to TAVI under normal circumstances. We assume that the effect of TIM in cardiovascular patients, which is established before TAVI, persists up to 6 months after the heart team's decisions. Thus, we hypothesize that days lost to cardiovascular hospitalization and all-cause mortality will be sustainably reduced by TIM compared to standard care up to 6 months after the heart team's decision (primary endpoint). In addition to hospitalization and mortality, we will also assess the quality of life, and expect patients in the TIM group to report fewer symptoms and greater well-being due to intensive outpatient care and trained self-reliant management. TIM interventions, including adjustment or prescription of medication, recommendation of hospital admissions, and patients’ adherence to TIM, will be reported as secondary endpoints. However, the telemedicine center comprises a team of four physicians and heart failure nurses with 8 working hours per day. Therefore, a requirement for the successful integration of TIM into daily clinical practice is the reimbursement by the healthcare system.

Few studies investigated TIM in AS patients undergoing TAVI. The studies focused mainly on the detection of conduction disturbances after TAVI and the need for pacemaker implantation (19, 20). In the prospective cohort study Redirect TAVI, continuous pocket electrocardiograms were used to detect conduction disturbances in 192 AS patients without pacemakers 14 days before and after TAVI (19). Another study used a wearable smartwatch-facilitated remote health management including assessment of heart rate, rhythm, oxygen saturation, and activity in 100 patients with AS 1 day before to 30 days after TAVI discharge (20). Both cohort studies detected relevant arrhythmias leading to pacemaker implantation in 4%–5% of patients post-TAVI; wearable smartwatch-facilitated management enabled additional telemedicine interventions such as a change in medical treatment in 5% of patients (19, 20). In contrast, we plan to use TIM in the waiting period before admission for TAVI compared with standard care. We expect to reduce days lost due to cardiovascular hospitalization and all-cause mortality before TAVI and according to the sustained effect of TIM until 180 days after the heart team's decision.

A potential limitation of the ResKriVer-TAVI study may be the variable and sometimes short period of TIM in patients with AS who are planned for TAVI.

In conclusion, the ResKriVer-TAVI study will be the first randomized trial to evaluate the efficiency of TIM in AS patients who are planned for TAVI. Results are awaited in 2025.
